# Structural Analysis Implicates CASK-Liprin-α2 Interaction in Cerebellar Granular Cell Death in MICPCH Syndrome

**DOI:** 10.3390/cells12081177

**Published:** 2023-04-18

**Authors:** Qi Guo, Emi Kouyama-Suzuki, Yoshinori Shirai, Xueshan Cao, Toru Yanagawa, Takuma Mori, Katsuhiko Tabuchi

**Affiliations:** 1 Department of Molecular and Cellular Physiology, Shinshu University School of Medicine, Matsumoto 390-8621, Japan; 2 College of Life Science and Oceanography, Shenzhen University, Shenzhen 518071, China; 3 Department of Oral and Maxillofacial Surgery, Faculty of Medicine, University of Tsukuba, Tsukuba 305-8575, Japan; 4 Department of NeuroHealth Innovation, Institute for Biomedical Sciences, Interdisciplinary Cluster for Cutting Edge Research, Shinshu University, Matsumoto 390-8621, Japan

**Keywords:** MICPCH syndrome, CASK, Liprin-α2, cerebellar granule cells, CaMK domain

## Abstract

Microcephaly with pontine and cerebellar hypoplasia (MICPCH) syndrome is a neurodevelopmental disorder caused by the deficiency of the X-chromosomal gene CASK. However, the molecular mechanisms by which CASK deficiency causes cerebellar hypoplasia in this syndrome remain elusive. In this study, we used CASK knockout (KO) mice as models for MICPCH syndrome and investigated the effect of CASK mutants. Female CASK heterozygote KO mice replicate the progressive cerebellar hypoplasia observed in MICPCH syndrome. CASK KO cultured cerebellar granule (CG) cells show progressive cell death that can be rescued by co-infection with lentivirus expressing wild-type CASK. Rescue experiments with CASK deletion mutants identify that the CaMK, PDZ, and SH3, but not L27 and guanylate kinase domains of CASK are required for the survival of CG cells. We identify missense mutations in the CaMK domain of CASK derived from human patients that fail to rescue the cell death of cultured CASK KO CG cells. Machine learning-based structural analysis using AlphaFold 2.2 predicts that these mutations disrupt the structure of the binding interface with Liprin-α2. These results suggest that the interaction with Liprin-α2 via the CaMK domain of CASK may be involved in the pathophysiology of cerebellar hypoplasia in MICPCH syndrome.

## 1. Introduction

Calcium/calmodulin-dependent serine protein kinase (CASK) is a membrane-associated guanylate kinase (MAGUK) protein originally identified as an intracellular binding partner for Neurexins [1]. Since Neurexins are presynaptic cell adhesion proteins, CASK has been initially considered to function for presynaptic formation, but later it turned out that CASK also acts for postsynaptic maturation or neural development through interaction with multiple proteins through its functional domains [2,3,4]. The CASK protein consists of a CaMK domain, two tandem L27 domains, a PDZ domain, an SH3 domain, and a guanylate kinase domain from the N- to C-terminal [5]. At the presynaptic terminal, CASK functions as a hub for the Neurexin complex. CASK binds Neurexins at multiple sites encompassing PDZ, SH3, and guanylate kinase domains [1]. Mint1, Caskin1, Tiam1, and Liprin-α competitively bind to the CaMK domain, suggesting that they share the common binding site in the CaMK domain [6,7,8,9,10,11]. Liprin-α also binds to CASK at an additional site in a noncompetitive manner with Mint1 in the presence of Neurexin [12]. The CaMK domain of CASK has been shown to phosphorylate three serines in the cytoplasmic tail of Neurexin1 at low concentrations of divalent cations, such as magnesium [13]. These serine phosphorylations destabilize CASK-Neurexin-Liprin-α complex and dissociate Liprin-α from the complex [12]. CASK functions as a transcriptional regulatory factor in cooperation with T-Box brain transcription factor 1 (Tbr1) [3]. CASK binds to Tbr1 through the C-terminal guanylate kinase domain [3]. The CASK-Tbr1 complex is translocated to the nucleus and promotes the transcription of downstream target genes, such as Grin2B and Reelin [14,15].

CASK is encoded by the gene on the X-chromosome in mammals. CASK knockout (KO) mice exhibit cleft palates resulting in perinatal lethality due to feeding difficulties or respiratory failure [16]. CASK KO cortical culture neurons show an imbalance between excitatory and inhibitory synaptic transmission [16]. Mutations or deletions in the CASK gene have been discovered in patients with neurodevelopmental disorders, including X-linked intellectual disability (XLID), autism, infantile spasms, nystagmus, optic atrophy, and FG syndrome [17,18,19,20,21,22]. Microcephaly with pontine and cerebellar hypoplasia (MICPCH) syndrome is the severest form of CASK deficient disorder in which many of these symptoms are associated with microcephaly and pontocerebellar hypoplasia [23,24,25]. Most of the patients with MICPCH syndrome are women. Considering the fact that male CASK KO mice are perinatally lethal, males with CASK deficiency may result in a miscarriage before birth. Male patients with MICPCH syndrome caused by CASK somatic mosaic deficiency have also been reported [26,27]. The X-chromosome inactivation mechanism has been shown to be involved in the pathophysiology of the brain phenotype in heterozygote female CASK KO mice [28]. CASK positive and negative neurons are randomly distributed in the cerebral cortex of CASK heterozygote female KO mice [28]. The expression level of Grin2B is decreased in CASK deficient neurons due to a lack of CASK-Tbr1 transcriptional function [28]. This results in an increase in excitatory and a decrease in inhibitory synaptic inputs into CASK-deficient neurons in CASK heterozygote female KO mice [28]. These changes are undetectable in the synapses projecting onto CASK wild-type neurons in CASK heterozygote female KO mice [28]. Excitatory and inhibitory imbalanced synaptic inputs are not observed in conditional CASK KO neurons in which CASK is removed after brain maturation, suggesting that these phenotypes are due to the developmental effects of CASK deficiency [28]. The phenotypes observed in the cerebral cortex in CASK KO mice may be attributable to social deficits, intellectual disability, and infantile spasms in MICPCH syndrome. However, the pathophysiology underlying cerebellar hypoplasia, which is the most prominent phenotype of MICPCH syndrome, remains elusive.

Loss of cerebellar granular (CG) cells has been shown to be the main pathology in cerebellar hypoplasia in MICPCH syndrome. Experimental studies utilizing cell-type and developmental stage-specific CASK conditional KO mice have revealed that the deficiency of CASK affects the degeneration of CG cells rather than the layer formation of the cerebellum [29,30]. Furthermore, mutations in CASK that have been identified in MICPCH syndrome patients with cerebellar hypoplasia have been reported to reduce the binding affinity of CASK to Liprin-α2 [31]. However, the relevance between CASK-Liprin-α2 interaction and the degeneration of CG cells has not been investigated.

To investigate the molecular mechanism by which CASK deficiency causes cerebellar hypoplasia, we examined the effects of CASK mutations on the survival of CG cells in mice. We identified some human-patient-derived missense mutations within the CaMK domain of CASK that failed to rescue cultured CG cell death in CASK KO mice. In silico structural analysis using machine learning-based software revealed that these missense mutations disrupt the binding interface with Liprin-α2, suggesting that CASK-Liprin-α2 interaction may be involved in the pathophysiology of cerebellar hypoplasia in MICPCH syndrome.

## 2. Materials and Methods

### 2.1. Animals and Housing Conditions

All animals were group-housed, maintaining a 12:12 light-dark cycle (lighting from 09:00 to 21:00) with food and water ad libitum. The room temperature was maintained at 23 ± 2 °C. All procedures of animal experiments were reviewed by the Committee for Animal Experiments and were finally approved by the president of Shinshu University. The methods were carried out in accordance with the Regulations for Animal Experimentation of Shinshu University.

### 2.2. Genotyping

Genomic DNA was prepared from clipped ears by digesting in 25 mM NaOH at 95 °C for 20 min, vortexed, and neutralized with 40 mM Tris-HCl (pH 8.0). Genotyping of CASK floxed/floxed was performed using primers KT12286 and KT12287, CASK floxed/floxed and CASK floxed/Y was performed using KT12287 and KT12288. The PCR condition was as follows. One cycle of 94 °C for 3 min, followed by 40 cycles of 98 °C for 10 s, 65 °C for 30 s, 68 °C for 10 s, using KOD FX Neo polymerase (TOYOBO).

### 2.3. Plasmid Construction

pFSy-ratSyn-Flag-CASK (WT): Total rat brain RNA extraction was described elsewhere [32]. In short, the total RNA of the rat forebrain (Wistar, 6-week-old male) was isolated by ISOGEN (Takara, Shiga, Japan) and cDNAs were obtained using SuperScript II reverse transcriptase (Invitrogen, Waltham, MA, USA) and Oligo(dT) primer following the manufacturer’s instructions. Flag-tagged CASK was amplified from rat brain cDNAs using PrimeSTAR Max DNA polymerase (Takara, Shiga, Japan), and a primer pair A. EGFP was removed by digestion with BamHI and EcoRI from pFSy (1.1) GW (Addgene #27232). A 2.8 kbp DNA fragment coding Flag-CASK was digested by BclI and EcoRI, then cloned into the BamHI/EcoRI site of pFSy (1.1) GW using Ligation high ver.2 (TOYOBO). The primers used for sequencing were listed in the primer table. pFSyGW-iCre: BamHI-iCre-EcoRI was cloned into the BamHI/EcoRI site of pFSy (1.1) GW. pFSyGW-control: pFSyGW-iCre was digested by BamHI and EcoRI, blunt-ended by Klenow fragment (Takara, Shiga, Japan), and self-ligated. Lenti vectors for the expression of CASK deletion mutants were constructed by mutating pFSy-ratSyn-Flag-CASK (WT) using PrimeSTAR Mutagenesis Basal Kit (Takara, Shiga, Japan) following the manufacturer’s instruction. The sets of primers used for mutagenesis were (ΔCaMK_F/ΔcaMK_R), (ΔL27_F/ΔL27_R), (ΔPDZ_F/ΔPDZ_R), (ΔSH3_F/ΔSH3_R) and (ΔGK_F/ΔGK_R); for pFSy-ratSyn-Flag-CASK (ΔcaMK),pFSy-ratSyn-Flag-CASK (ΔL27),pFSy-ratSyn-Flag-CASK (ΔPDZ), pFSy-ratSyn-Flag-CASK (ΔSH3), pFSy-ratSyn-Flag-CASK (ΔGK), respectively. Lenti vectors for the expression of CASK missense mutants derived from human patients were constructed by mutating pFSy-ratSyn-Flag-CASK (WT) using PrimeSTAR Mutagenesis Basal Kit (Takara, Shiga, Japan) following the manufacturer’s instruction. Primers used for mutagenesis were (R28L_F/R28L_R), (D58E_F/D58E_R), (R106P_F/R106P_R), (G197R_F/G197R_R), (L209P_F/L209P_R), (R255C_F/R255C_R), (Y268H_F/Y268H_R), (P625L_F/P625L_R), (G637D_F/G637D_R), (R489W_F/R489W_R), (M507I_F/M507I_R), (M519T_F/M519T_R), (R521V_F/R521V_R), (G659D_F/G659D_R); for pFSy-ratSyn-Flag-CASK (R28L), pFSy-ratSyn-Flag-CASK (D58E), pFSy-ratSyn-Flag-CASK (R106P), pFSy-ratSyn-Flag-CASK (G197R), pFSy-ratSyn-Flag-CASK (L209P), pFSy-ratSyn-Flag-CASK (R255C), pFSy-ratSyn-Flag-CASK (Y268H), pFSy-ratSyn-Flag-CASK (P625L), pFSy-ratSyn-Flag-CASK (G637D), pFSy-ratSyn-Flag-CASK (R489W), pFSy-ratSyn-Flag-CASK (M507I), pFSy-ratSyn-Flag-CASK (M519), pFSy-ratSyn-Flag-CASK (R489W), pFSy-ratSyn-Flag-CASK (G659D), respectively. Note that human R28, D58, R106, G197, L209, R255, Y268, R489, M507, M519, and R521 are conserved (both amino acid and nucleic acid) between humans and rats. However, the human P625L, G637D, and G659D missense mutants correspond to rat P623L (1838 C > T), G625D (1874 G > C), and G647D (1940 G > A), respectively. For the expression of EGFP-CASK fusion proteins in HEK cells, pCMV-EGFP-CASKs (WT, deletion mutants, and missense mutants) were constructed as follows. PCR products coding CASK cDNAs (WT or mutants) containing the BsrGI site at their 5’end and the XbaI site at their 3’end were amplified using a primer set (EGFP_CASK_InFusion_F/EGFP_CASK_InFusion_R) from each pFSy-ratSyn-CASK plasmid (WT or mutants) as a template. PCR fragments were cloned into the BsrGI/XbaI site of pEGFP-N3 (Clontech, Palo Alto, CA, USA) using InFusion HD Cloning Kit (Takara, Shiga, Japan). pCMV-Myc-Liprin α2: Myc-tagged Liprin a2 was amplified from rat brain cDNAs using PrimeSTAR Max DNA polymerase and a primer pair for cloning Myc-Liprin α2. EGFP was removed by digestion with BamHI and EcoRI from pEGFP-N3 (Clontech, Palo Alto, CA, USA). A 3.6 kbp DNA fragment coding Myc tagged Liprin α2 was cloned into the BamHI/EcoRI site of pEGFP-N3 using InFusion HD Cloning Kit (Takara, Shiga, Japan).

### 2.4. Cell Lines

HEK293T cells and Lenti-X 293T cells (Takara, Shiga, Japan) were treated at 37°C in a 5% CO_2_ atmosphere to maintain saturation in Dulbecco’s modified Eagle’s Medium (DMEM) (ThermoFisher Scientific, Waltham, MA, USA) supplemented with 5% FBS (Biowest, Nuaillé, France) and 100 U/mL of Antibiotic-Antimycotic Mixed (Nacalai tesque, Kyoto, Japan).

### 2.5. Lentiviral Preparation

Lentivirus preparations were described elsewhere [33]. In short, HEK cells were transfected with each lentiviral plasmid, pMDLg/pRRE, pRSV-Rev, and pMD2.G plasmids. Forty-eight hours after transfection, the lentiviruses were harvested, concentrated using the Lenti-X concentrator (Takara, Shiga, Japan), resuspended with PBS, divided into aliquots, and stored at −80 °C.

### 2.6. Cell Culture and Immunocytochemistry

Primary CG cell cultures were prepared from CASK floxed/floxed, Nrxn123 floxed/floxed, and wild-type C57BL/6 at P5, as described previously [33]. In short, isolated cerebella were incubated with phosphate-buffered saline (PBS) containing 1% trypsin (Sigma-Aldrich, St. Louis, MO, USA) and 0.1% DNase I (Sigma-Aldrich) for 3 min and dissociated by passing through a fire-polished Pasteur pipette in PBS containing 0.05% DNase I, 0.03% trypsin inhibitor (Sigma-Aldrich, St. Louis, MO, USA), and 2 mM MgCl_2_. Cells were plated on coverslips coated with poly-L-lysine (Sigma-Aldrich, St. Louis, MO, USA) and mouse laminin (ThermoFisher Scientific, Waltham, MA, USA) at a density of 2 × 10^5^ cells/well or 3 × 10^5^ cells/well. Cells were cultured in Neurobasal-A (ThermoFisher Scientific, Waltham, MA, USA) supplemented with 2% B-27 supplement (ThermoFisher Scientific, Waltham, MA, USA), 5% fetal bovine serum (FBS) (Biowest, Nuaillé, France), 100 U/mL of Antibiotic-Antimycotic Mixed (Nacalai tesque, Kyoto, Japan), and 2 mM GlutaMAX I (ThermoFisher Scientific, Waltham, MA, USA) for 24 h, and then cultured in the same medium without FBS. The cultured CG cells were incubated at 37 °C in a 5% CO_2_ atmosphere saturated with water. To confirm CASK expression of cultured CG cells in fCASK, cultured cells were infected with either an empty lentivirus (pFSy(1.1)GW) as a control or lentivirus-iCre (pFSyGW-iCre) at DIV1. The cultured cells were fixed with 4% PFA/4% sucrose in PBS at DIV5 and immunostained with rabbit anti-CASK (Abcam, Cambridge, UK, 1:50) and mouse anti-tubulin3/Tuji1 (Genetex, Irvine, CA, USA, 1:1000) antibodies, followed by incubation with Donkey anti-rabbit IgG H&L (Alexa Fluor^®^ 594) (Abcam, Cambridge, UK, 1:500) and Donkey anti-Mouse IgG H&L (Alexa Fluor^®^ 647) (Abcam, Cambridge, UK, 1:500). For the CG cells survival, the cultured cells were infected with either an empty lentivirus (pFSy(1.1)GW) or the lentivirus-iCre (pFSyGW-iCre) at DIV1. Cultured cells were fixed at DIV3, 5, 7, or 9 and immunostained with a Mouse Anti-NeuN antibody (Sigma-Aldrich, MAB377, 1:2000), followed by incubation with Donkey anti-Mouse IgG H&L (Alexa Fluor^®^ 594) (Abcam, Cambridge, UK, ab150108, 1:1000). For double infection, the cultured CG cells were first infected with empty lentivirus (pFSy(1.1)GW) or lentivirus (pFSyGW-iCre) expressing iCre for 3 h at DIV1. After the first infections, the culture medium was replaced with fresh medium, followed by infection with an empty lentivirus (pFSy(1.1)GW) and a lentivirus expressing CASK or mutant CASKs. The cultured cells were fixed at DIV9 and immunostained with a Mouse Anti-NeuN antibody. For treatment with neurotrophic factors, 40 ng/mL of human BDNF (Sigma-Aldrich) was applied to the cultured CG cells at DIV2. The cultured cells were fixed with 4% paraformaldehyde (PFA) and 4% sucrose in PBS at DIV9 and immunostained with a Mouse Anti-NeuN antibody. Fluorescence images were acquired with a confocal laser scanning microscope (TCS SP8; Leica Microsystems, Wetzlar, Germany). CG cells were identified based on NeuN signals and their morphological features, small size (5–10 μm in diameter), regular round or ovoid shape [34].

### 2.7. TUNEL Assay

Terminal deoxynucleotidyl transferase-mediated dUTP nick end labeling (TUNEL) assay was performed using the in situ Cell Death Detection kit, Fluorescein (Roche, Basel, Switzerland), according to the instructions of the manufacturer. The cultured fCASK CG cells were infected with either an empty lentivirus (pFSyGW-control) or the lentivirus expression iCre (pFSyGW-iCre) for 3 h at DIV1. After the first infection, the culture media were replaced with fresh media, followed by infection with an empty lentivirus (pFSyGW-control) and a lentivirus expressing CASK or mutant CASKs. The CG cells were fixed at DIV7 and incubated in 0.25% Triton X-100 (Nacalai tesque, Kyoto, Japan) at RT for 5 min, followed by incubation with reaction solution at 37 °C for 60 min and stained with DAPI. Fluorescence images were acquired with a confocal laser scanning microscope (TCS SP8; Leica Microsystems, Wetzlar, Germany).

### 2.8. Immunoblotting

Cultured CG cells were solubilized with RIPA buffer (20 mM HEPES pH 7.4, 100 mM NaCl, 1 mM EDTA, 1% Triton-X 100 containing the protease inhibitor cocktail (Nacalai tesque, Kyoto, Japan). The protein concentration of the CG cell lysate was quantified by the BioRad Protein Assay System (BioRad, Berkeley, CA, USA). Twenty μg protein was subjected to SDS-PAGE (7.5% Laemmli) and electroblotted onto Immobilon-FL PVDF membrane (Millipore, Burlington, MA, USA). Western blot was performed using anti-Flag mouse monoclonal antibody (MBL, M185-3S, 1:1000), anti-CASK polyclonal antibody (Abcam, Cambridge, UK, ab3383, 1:1000), anti-CASK monoclonal antibody (NeuroMab, K56A/50, 1:1000), anti-β-Actin mouse anti-Actin monoclonal antibody (MBL, Tokyo, Japan, M177-3, 1:1000), IRDye 800 CW goat anti-rabbit secondary antibody (LI-CDR Bioscience, Lincoln, NE, USA, 1:20,000), IRDye 800 goat anti-Mouse secondary antibody (LI-CDR Bioscience, Lincoln, NE, USA, 1:20,000) and visualized by ODYSSEY Imaging System (LI-CDR Bioscience, Lincoln, NE, USA).

### 2.9. Coimmunoprecipitation

Coimmunoprecipitation was performed following the method described elsewhere (Tibbe et al., 2022). In short, HEK293T cells were transfected with CASK mutant plasmid, pEGFP N3 (negative control), pCMV-EGFP-CASK(WT), pCMV-EGFP-CASK(R106P), pCMV-EGFP-CASK(R255C), or pCMV-EGFP-CASK(Y268H), respectively. Each group was co-transfected with the plasmid pCMV-Myc Liprin α2. Twenty-four hours after transfection, cells were harvested and lysed with lysis buffer (20 mM Tris-HCl pH 7.5, 150 mM NaCl, 1.0 mM EDTA, 1% Triton X-100 containing protease inhibitors cocktail (Nacalai tesque, Kyoto, Japan). Lysates were subjected to immunoprecipitation using GFP-Trap Agarose (ChromoTek GmbH, Martinsried, Germany), or Anti-c-Myc Antibody Beads (10D11) (FUJIFILM Wako, Osaka, Japan). Immunoprecipitate (IP) and input samples (Input) were subjected to Western blotting using anti-CASK monoclonal antibody (NeuroMab Facility, Davis, CA, USA, K56A/50, 1:2000), anti-c-Myc monoclonal antibody (FUJIFILM Wako, Osaka, Japan, 10D11, 1:500), and IRDye 800 anti-mouse fluorescent secondary antibody (LI-CDR, Lincoln, NE, USA, 1:5000). Protein bands were detected by Odyssey Imaging System (LI-CDR, Lincoln, NE, USA). Band intensities were quantified using ImageJ software (version 2.3.0, FIJI, NIH, USA).

### 2.10. RT-qPCR

The expression level of the target genes was quantified by qPCR using GAPDH as an endogenous control. In short, mice CG cell cultures were infected with lentivirus (pFSy(1.1)GW) or lentivirus pFSyGW-iCre at DIV1 and harvested at DIV5. Total RNA was extracted using TRIzol (Ambion, Carlsbad, CA, USA) following the manufacturer’s instructions. One ug RNA was converted to cDNA using the High-Capacity cDNA Reverse Transcription Kit (Applied Biosystems, Foster City, CA, USA). Fifty ng cDNA was subjected to qPCR using Power SYBR Green PCR Mix (Applied Biosystems, Warrington, UK) on QuantStudio3 (ThermoFisher Scientific, Waltham, MA, USA). The primer pairs used were BDNF-F/BDNF-R (for BDNF), Casp-F/Casp-R (for Caspase-3), p38-F/p38-R (for p38), and KT10045/KT10046 (for GAPDH). The PCR condition was as follows: 50 °C for 20 min and 95 °C for 10 min, followed by 40 cycles of 95 °C for 15 s and 60 °C for 1 min. The melt curve stage was 95 °C for 15 s, 60 °C for 1 min, and 95 °C for 15 s. The fold changes of BDNF, Caspase-3, and p38 mRNA were calculated by the delta-delta Ct method.

### 2.11. Hematoxylin & Eosin Staining

P6 and P14 mice were transcranial perfused with 4% PFA in PBS. Brains were removed, postfixed in 4% PFA, 24 h later put in the 15% sucrose in PBS, 24 h later put in the 30% sucrose, embedded in Tissue Tek compound, and stored at −80 °C. Successive 20 µm thick sagittal sections were prepared using a cryostat microtome (Leica Microsystems, Wetzlar, Germany, CM1950), and stained with hematoxylin and eosin (H&E). Images were acquired with a Keyence microscope (Keyence BZ-X810). Images of the whole cerebellum were taken using a 40× lens, and CG regions were taken using a 100× lens. The CG cell density (cells/area) in cerebellum lobules IV/V and IX area were analyzed using ImageJ software (version 2.3.0, FIJI, NIH, USA).

### 2.12. Intracellular Localization of CASK Mutants

We constructed plasmids pCMV-EGFP-CASKs and pCAG-tagRFP, then transfected HEK-293T cells using lipofectamine 2000 Reagent (Invitrogen). Intracellular localization of EGFP-CASKs was investigated by fluorescence signals EGFP using a confocal laser scanning microscope (Leica Microsystems, Wetzlar, Germany, TCS SP8).

### 2.13. Prediction of CASK Protein Structure and Structural Analysis

We predicted the protein structure of the CaMK domain of CASK. The original sequence of the amino acids in the CaMK domain of CASK was obtained from the PDB database (6LNM), which has a shorter additional sequence on the N-terminal of CASK. Since the predicted full length of CASK did not match any of the reported structural models of the CaMK domain of CASK in our preliminary experiments, we used the amino acid sequence in the PDB structural model as a template. We then introduced one of the amino acid substitutions of the CASK missense variants used in this study. Sequences that included one of the point mutations were used as a query sequence in ColabFold (https://colab.research.google.com/github/sokrypton/ColabFold/blob/main/AlphaFold2.ipynb (accessed on 19 December 2022)) to predict protein structure using the default settings. We selected model 4 among the five generated models because this model was ranked in the top 2 in all the predictions. The PDB files obtained by the model were used for further analysis on PyMOL (Schrödinger and DeLano (2020). PyMOL. Retrieved from http://www.pymol.org/pymol, accessed on 19 December 2022).

For the evaluation of the predicted CASK, we superimposed it on the CASK structure (3C0H). For the evaluation of the interaction between CASK and Liprin-α2/Mint1, the structural model between CASK and Liprin-α2 (3TAC) or between CASK and Mint1 (6LNM) was opened on PyMOL and a PDB file of one of the predicted CASK variants was loaded. Then, one of the predicted CASK variants was aligned to the CASK in the model using the ‘align’ function of PyMOL. To detect hydrogen bonds, we selected the target amino acid on CASK and used the ‘find’ function of PyMOL.

### 2.14. Sample Size and Statistical Analysis

Prism 9 (GraphPad) was used for all statistical analyses. Statistical tests performed in this study were Student’s two-tailed t-test, One-way ANOVA and Two-way ANOVA. *p*-values of the relevant post hoc analyses are shown in the figures. The statistical significance cutoff was set at *p* < 0.05, and the data are presented as means ± SEM of the mean. F-statistics and *p* values are stated in the figure legends, and relevant post hoc comparisons are shown in the figures. n ≥ 3 mice were used for each experiment, and the sample size for each experiment is stated in the figure legends and the text.

## 3. Results

### 3.1. Density of CG Cells Is Decreased in CASK+/− Mice

**Figure 1:**
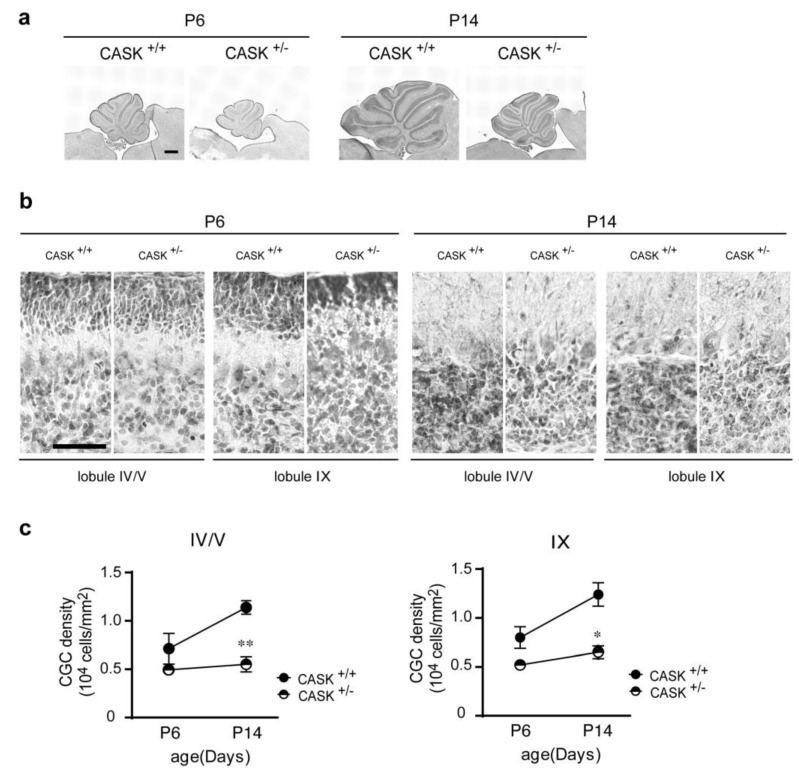
Progressive loss of CG cells in heterozygous CASK-KO mice. (**a**) Developmental neuroanatomical changes of the cerebella of wild-type (CASK+/+) and heterozygous CASK-KO (CASK+/−) mice. Parasagittal cerebellar sections prepared from 6- and 14-day-old wild-type and heterozygous CASK-KO mice are stained with hematoxylin. Scale bars = 500 μm. (**b**) Magnified images of the cerebellar cortex of 6- and 14-day-old mice in a. Lobules IV/V and IX are shown. Scale bar = 50 μm. (**c**) Quantification of developmental changes in CG cell densities in control and heterozygous CASK-KO mice. All values represent the mean ± s.e.m., n = 3 each from three animals. ** *p* < 0.01, * *p* < 0.05; Student *t*-test.

CASK female heterozygote KO mice exhibit shrinkage of cerebellum associated with ataxic phenotype [28,29]. To investigate the morphological abnormalities in-depth, we prepared hematoxylin-eosin-stained tissue slices containing cerebellum at two different ages, post-natal day 6 (P6) and 14 (P14), in wild-type (CASK+/+) and CASK female heterozygote KO (CASK+/−) mice and analyzed on light microscopy. Parasagittal sections of these mice indicate that the overall size of the cerebellum was reduced in CASK+/− at both P6 and P14 compared to CASK+/+ mice (Figure 1a). In CASK+/+ mice, the density of CG cells was increased from P6 to P14 in lobule IV/V (1.599-fold) and IX (1.550-fold) (Figure 1b,c). On the other hand, that in CASK+/− was smaller and not increased as CASK+/+ from P6 to P14 (1.117-fold in IV/V and 1.247-fold in IX) (Figure 1c).

### 3.2. Apoptosis Occurs in CASK KO CG Cells

**Figure 2:**
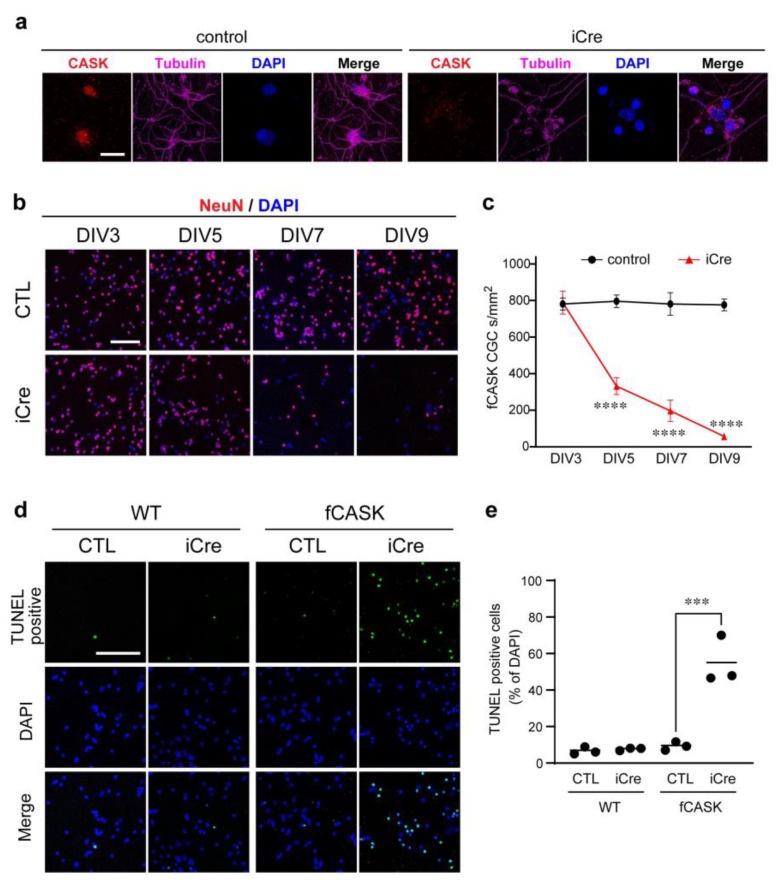
CASK is essential for the survival of cultured CG cells. (**a**) Lentivirus-iCre-mediated CASK KO in cultured CG cells. Cultured homozygote floxed CASK (fCASK) CG cells were infected with lentivirus-iCre at DIV1 and stained with antibodies against CASK and tubulin at DIV7. The CASK signal is absent in iCre-infected CG cells. Scale bars = 20 µm. (**b**) Cultured fCASK CG cells were infected with lentivirus-iCre. The cells are double stained with the antibody against NeuN (red) and DAPI (blue). (**c**) Quantification of the number of NeuN positive CG cells in (**b**) (n = 9 each). **** *p* < 0.0001; two-way ANOVA. (**d**) TUNEL analysis of CG cells. Cultured wild-type (WT) or fCASK CG cells are infected with lentivirus-iCre. Cells are stained using the In situ Cell Death Detection Kit (green) and DAPI (blue). Scale bar = 100 µm. (**e**) Quantification of TUNEL-positive cells in (**d**). All values present the mean. Each dot in the graph represents the average density of randomly selected three areas in each well. n = 3 (wells) in each group. Five to eight mouse brains were collected for preparing CG cell cultures to aliquot into each well. *** *p* < 0.001; one-way ANOVA.

To investigate the effect of CASK deficiency on cell death in CG cells, we established a CG cell dissociated culture [33]. For this, we prepared CG cell cultures from CASK homozygote floxed mice and infected with lentiviruses expressing iCre recombinase under the control of the rat synapsin1 promoter and generated CASK KO CG cells. CASK floxed CG cells infected with empty lentiviruses were used as control. We confirmed that immunoreactivity to the CASK protein was absent in iCre-infected floxed CG cell cultures (Figure 2a). To investigate the time course of cell death, we quantified the density of CG cells at DIV3, DIV5, DIV7, and DIV9 in control and CASK KO cultures by immunostaining with NeuN antibody, a marker for CG cells. The density of CG cells was progressively decreased in CASK KO CG cells (Figure 2b,c).

To examine whether CG cell death is due to apoptosis or not, we performed a TUNEL assay on CG cell cultures. We compared TUNEL signals between control and iCre-infected CG cell cultures from wild-type and floxed CASK mice at DIV7 (Figure 2d,e). TUNEL-positive CG cells were significantly increased in CASK KO CG cells, suggesting that cell death in CASK KO CG cells was caused by apoptosis (Figure 2d,e).

### 3.3. CG Cell Death in CASK KO Mice Is Independent of BDNF Secretion Mediated by Neurexins

**Figure 3:**
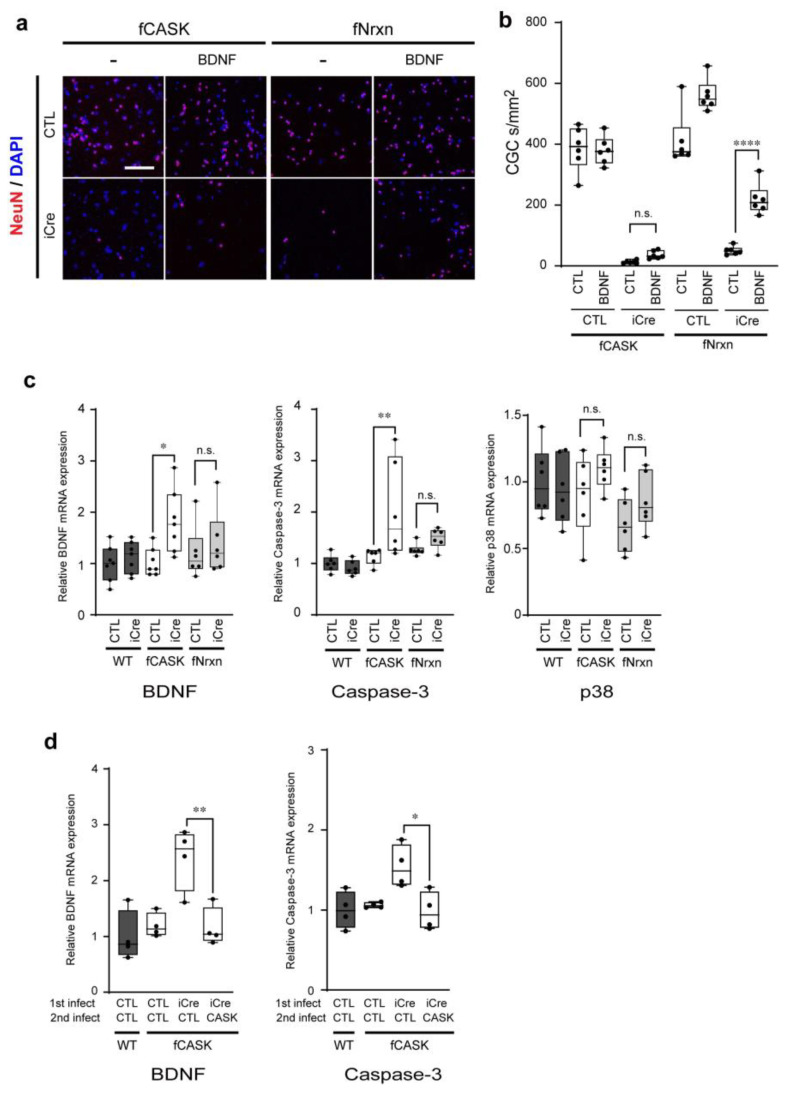
BDNF does not rescue cell survival defects in cultured CASK KO CG cells. (**a**) Cultured CG cells are prepared from homozygote fCASK and triple homozygote Neurexin-1, -2, -3 floxed (fNrxn) mice. BDNF is added to cultured CG cells infected with lentivirus control (CTL) or lentivirus-iCre (iCre). Cells are stained with the antibody against NeuN (red) and DAPI (blue). (**b**) Quantification of the number of NeuN-positive CG cells in a. Values are averages of 3 points for each well and 6 wells for each sample. **** *p* < 0.0001; one-way ANOVA. (**c**) Relative BDNF, caspase-3, and p38 expression levels in cultured CG CELLs as assessed by quantitative reverse transcriptase-PCR. Cultured CG cells are infected with lentivirus control (CTL) or lentivirus-iCre (iCre) at DIV1 and harvested at DIV5. The ratios of the mRNA expression levels of BDNF, caspase-3, or p38 to GAPDH are shown relative to that of WT CTL. All values present the mean ± s.e.m. (n = 6). (**d**) BDNF, and Caspase-3 mRNA expression in CASK KO CG cells rescued with lentiviral CASK infection. Cultured CG cells were infected with lentivirus control (CTL) or lentivirus-iCre (iCre) together with (CTL) or without CASK (CASK) expressing lentiviruses at DIV1 and harvested at DIV5. * *p* < 0.05, ** *p* < 0.01; one-way ANOVA, n.s. = not significant.

One of the representative functions of CASK is a scaffold of the Neurexin complex in the presynaptic terminal. Neurexin family proteins are transcribed by three genes (NRXN1, NRXN2, NRXN3). All three NRXNs produce longer α- and shorter β-forms [35,36,37], and NRXN1 additionally produces the shortest γ-form [38]. The last exons of all three neurexin genes encode transmembrane regions to cytoplasmic tails that are shared between α-, β-, and γ-forms. We previously generated Neurexin KO mice that remove all Neurexin isoforms by targeting the last exons of three Neurexins (Nrxn-TKO) [33]. Nrxn-TKO also shows cell death in CG cells in the culture system. This cell death was shown to be due to impaired secretion of brain-derived neurotrophic factor (BDNF). The application of BDNF in Nrxn-TKO culture rescues the cell death of CG cells [33]. To examine whether cell death of CASK KO CG cells is also attributable to depletion of BDNF, we applied BDNF in a CASK KO CG cell culture. In contrast to the Nrxn-TKO CG cell culture (fNrxn iCre BDNF) in which CG cell density was increased by the application of BDNF, the CG cell density was unchanged by BDNF application in CASK KO CG cell culture (fCASK iCre BDNF) (Figure 3a,b). We also examined the expression level of BDNF in CASK KO CG cells by quantitative RT-PCR. The transcription of BDNF was increased in CASK KO CG cells, while that was unchanged in Nrxn TKO (Figure 3c). The increase in BDNF expression in CASK KO CG cells was attenuated by reexpression of CASK by lentiviral infection in CASK KO CG cells (Figure 3d). However, the mechanism by which BDNF mRNA level is upregulated in CASK KO cells is unknown. We also examined the expression level of an apoptotic marker Caspase-3. The expression of Caspase-3 was increased in CASK KO, which was restored by reexpression of CASK (Figure 3c,d). This increase was not observed in Nrxn-TKO CG cells (Figure 3c). Both CASK KO and Nrxn-TKO CG cells did not change the expression of p38 (Figure 3c). These results suggest that the mechanism of cell death in CASK KO CG cells was distinct from that of Nrxn-TKO, and the Caspase-3 pathway is involved in the apoptotic mechanism in CASK KO CG cell death.

### 3.4. CaMK, PDZ, and SH3 Domains of CASK Are Required for the Survival of CG Cells

**Figure 4:**
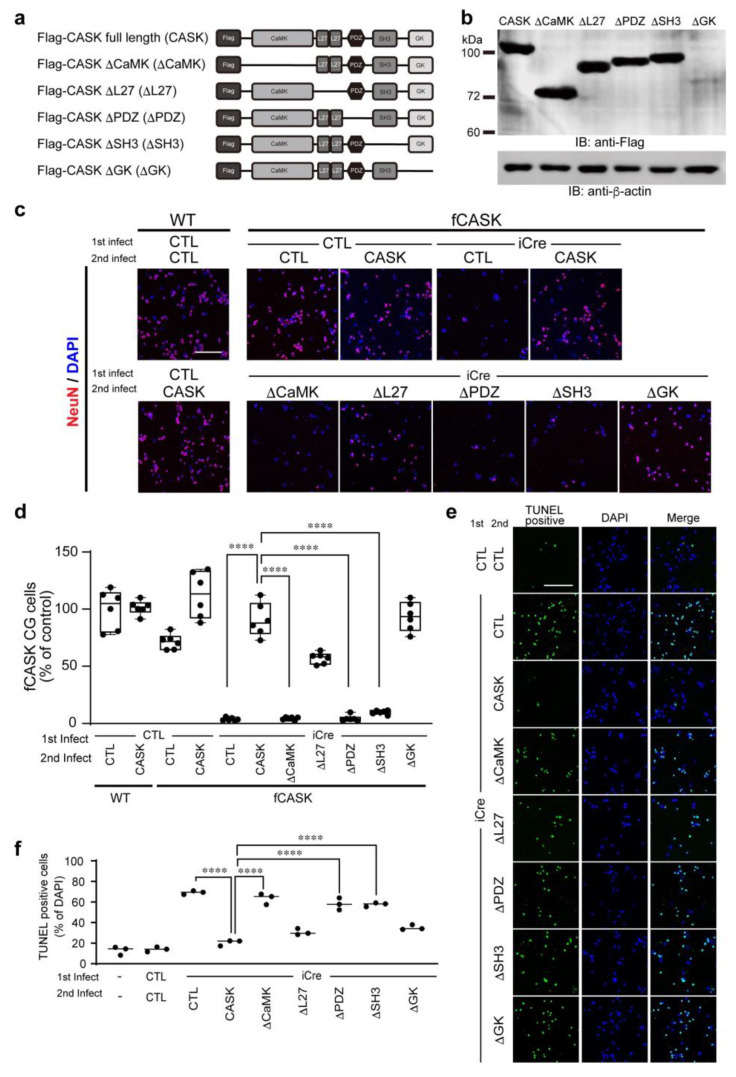
CASK deletion mutants affect the survival of cultured CG cells. (**a**) Schematic diagrams of full-length CASK and deletion mutants. (**b**) Western blot image for studying the expression of CASK deletion mutants. Cell lysates of WT CG cells infected with lentiviruses expressing the flag-tagged full-length and deletion mutant CASK are separated by SDS-PAGE, followed by Western blotting with anti-Flag and β-actin antibodies, respectively. (**c**) Effects of expression of CASK mutants on CASK KO CG cell survival. Cultured CG cells are infected with lentivirus-iCre (iCre) together with lentivirus-CASK (full-length or deletion mutants). Cells are stained with anti-NeuN antibody (red) and DAPI (blue). Scale bars = 100 µm. (**d**) Quantification of the number of NeuN-positive CG cells in (**c**). Each dot in the graph represents the average density of randomly selected three areas in each well. n = 6 (wells) in each group. Five to eight mouse brains were collected for preparing CG cell cultures to aliquot into each well. The horizontal line in each box indicates the median, the box shows the interquartile range (IQR), and the whiskers are the 1.5× IQR. **** *p* < 0.0001; one-way ANOVA. (**e**) TUNEL analysis of CG cells. Cultured CG cells are infected with lentivirus-iCre (iCre) together with lentivirus-CASK (full-length or deletion mutants). The cells are stained using the In situ Cell Death Detection kit (green) and DAPI (blue). Scale bars = 100 µm. (**f**) Quantification of TUNEL positive cells in (**e**). All values present the mean. Each dot in the graph represents the average density of randomly selected three areas in each well. n = 3 (wells) in each group. Five to eight mouse brains were collected for preparing CG cell cultures to aliquot into each well. **** *p* < 0.0001; one-way ANOVA.

To determine the functional domains of CASK that are responsible for the survival of CG cell, we performed rescue experiments in iCre infected fCASK CG cell cultures by co-infection with lentiviruses expressing full-length CASK or CASK deletion mutants that lack either CaMK (ΔCaMK), L27 (ΔL27), PDZ (ΔPDZ), SH3 (ΔSH3), or guanylate kinase (ΔGK) domains (Figure 4a). While ΔL27 and ΔGK, as well as full-length CASK, rescued the decreased density of CASK KO CG cells, ΔCaMK, ΔPDZ, and ΔSH3 failed to rescue it (Figure 4c,d). We confirmed that the ΔCaMK, ΔPDZ, and ΔSH3 constructs produced a significant amount of proteins by Western blot (Figure 4b) and immunocytochemistry (Supplementary Figure S4b). The expression level of ΔGK was weaker than others, but this construct rescued the CG cell density to the identical level of full-length CASK, indicating that this construct is functionally intact. In accordance with these results, apoptosis was increased in these unrescued CG cells (Figure 4e,f).

### 3.5. Missense Mutations Identified from Patients with Cerebellar Hypoplasia Affect CG Cell Survival

**Figure 5:**
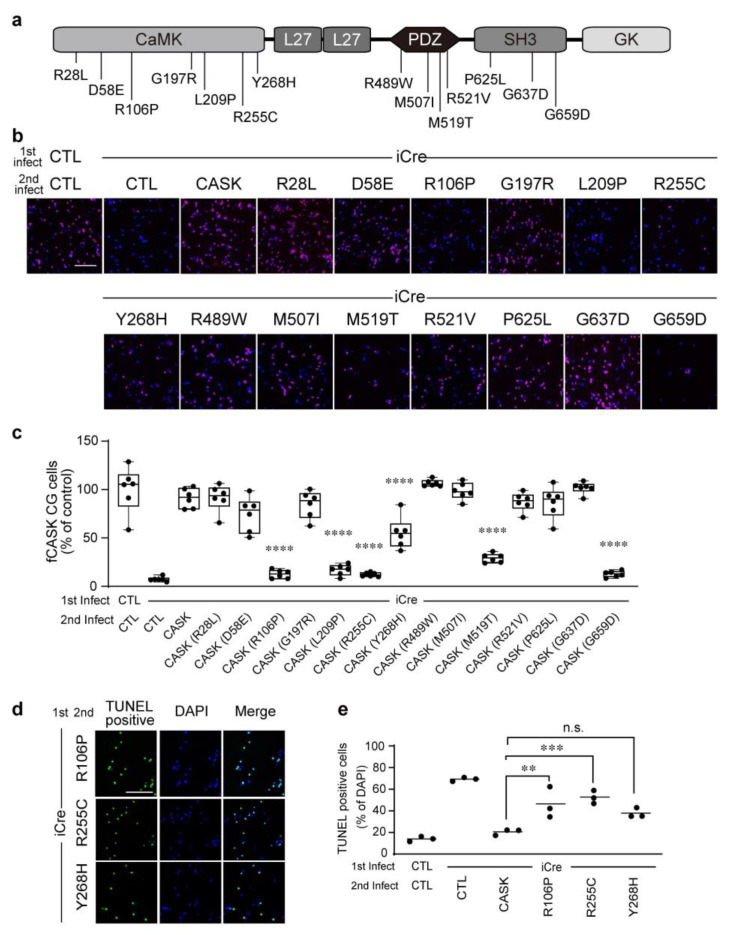
CASK missense mutations from human patients affect the survival of cultured CG cells. (**a**) Schematic diagram of CASK missense mutations identified from human patients. (**b**) Effects of CASK missense mutants on CASK KO CG cell survival. Cultured CG cells are infected with lentivirus-iCre (iCre) together with lentivirus-CASK (WT or mutants). Cells were stained with the antibody against NeuN (red) and DAPI (blue). Scale bars = 100 µm. (**c**) Quantification of the number of NeuN-positive CG cells in (**b**). The values are averages of 3 points for each well and 6 wells for each sample. The horizontal line in each box indicates the median, the box shows the interquartile range (IQR), and the whiskers are 1.5× IQR. **** *p* < 0.0001; one-way ANOVA. (**d**) TUNEL analysis of CG cells. Cultured fCASK CG cells are infected with lentivirus-iCre (iCre) together with lentivirus-CASK (WT or missense mutants; R106P, R255C, and Y268H). The cells are stained using the In situ Cell Death Detection kit (green) and DAPI (blue). Scale bars = 100 µm. (**e**) Quantification of TUNEL-positive cells in (**e**). All values present the mean ± s.e.m. The values are averages of 3 points for each well and 3 wells for each sample. ** *p* < 0.01, *** *p* < 0.001; one-way ANOVA, n.s. = not significant.

Although the results obtained from the deletion mutant experiments suggest the involvement of CaMK, PDZ, and SH2 domains in the mechanism underlying CG cell survival, it is important to note that such experiments have inherent limitations. It is possible that the deletion of each domain may impact the proper folding of the protein, thereby affecting the overall function of CASK. Furthermore, we cannot distinguish whether this is due to every single domain or super-domain effects. Therefore, we next examined the effect of CASK missense mutations identified in human patients manifesting neurological symptoms located in CaMK, PDZ, or SH3 domains. We generated lentiviral vectors for fourteen missense mutants, seven are in CaMK (R28L, D58E, R106P, G197R, L209P, R255C, and Y268H) [11,17,18,19,22,24,26,39,40,41], four are in PDZ (R489W, M507I, M519T, and R521V) [42,43], and three are in SH3 (P625L G637D, and G659D) [19,26,42,44] domains (Figure 5a). Co-infection with full-length and R28L, D58E, G197R, R489W, M507I, R521V, P625L, and G637D mutant forms of CASK rescued cell death in CASK KO CG cells (Figure 4b,c). On the other hand, R106P, L209P, R255C, Y268H, M521V, and G659 mutants failed to rescue cell death (Figure 4b,c). It may be possible that mutations that failed to rescue the CG cell death affect the proper expression of mutant forms of the CASK protein. We examined the expression levels of the mutant protein by Western blotting. In accordance with the previous report [40], we failed to detect a signal from L209P that has been shown to affect protein expression. On the other hand, we could successfully detect signals from R106P, R255C, Y268H, M521V, and G659D constructs in Western blot (Supplementary Figure S6a). Among these non-rescuing mutations, four are in the CaMK domain and three (R106P, R255C, and Y268H) are intact for their protein expression. To explore the common effect of these mutations may obtain a clue to understanding the molecular mechanism by which CASK deficiency affects CG cell survival. Thus, we decided to focus on these three mutations in CaMK domain in further analysis. We confirmed these three constructs (R106P, R255C, and Y268H) correctly expressed mutant proteins in HEK293 cells (Supplementary Figure S6b). As observed in CASK KO CG cells, the TUNEL signal was significantly increased in R106P or R255C co-infected CG cells, and not significantly but also increased in Y268H co-infected CG cells (Figure 5e). These results raised the hypothesis that the protein interaction via these mutation sites in CaMK domain may be critical for CG cell survival.

### 3.6. R106P, R255C, and Y268H Mutations Reside the Binding Interface between CASK-CaMK Domain and Liprin-α2

**Figure 6:**
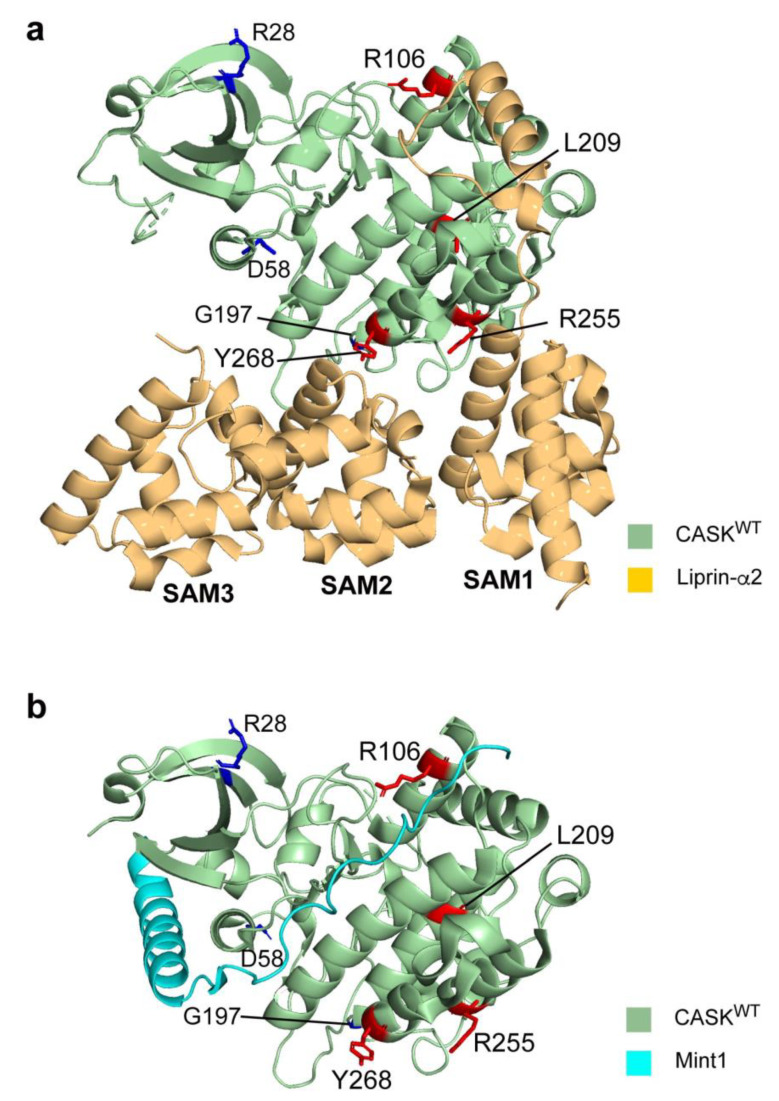
The missense mutations responsible for the survival of CGC are located in the binding interface between the CaMK domain of CASK and Liprin-α2 or Mint1. (**a**) Co-crystallography of the CASK-CaMK domain (green) and Lipin-α2 (orange) (3TAC). The positions of missense mutations that affect the survival of the CGC are shown in red (R106, L209, R255, and Y268), and those that do not affect are shown in blue (R28, G197, and D58). R106, R255, and Y268 are located at the binding interface between CASK-CaMK and Liprin-α2 (framed with an open square). (**b**) Co-crystallography between the CASK-CaMK domain (green) and Mint1 (sky blue) (6LNM). Only R106 is located in the biding interface between CASK-CaMK and Mint1.

To address the hypothesis that R106P, R255C, and Y268H mutations affect the protein interaction that is required for CG cell survival, we investigated the location of these mutations in the 3D structure of CASK-CaMK domain. A series of hydrophobic residues within the C-lobe of the CASK-CaMK domain constitutes a hydrophobic pocket where it interacts with hydrophobic motifs of Mint1 and Liprin-α2. R106P mutation is located near the hydrophobic pocket, likely affecting the interaction with these proteins [10,11]. CaMK domain of CASK interacts with Liprin-α2 at additional binding sites via the SAM domains of Liprin-α2 [10,11]. To verify the structural-functional relevance of these mutations, we mapped the mutation sites in cocrystallography models of the CASK-CaMK domain and the binding regions of these proteins. As previously reported, R106 is located at the binding interface between the hydrophobic pocket of the CASK-CaMK domain and the hydrophobic loop of Liprin-α2 and Mint1 (Figure 6). R255 and Y268 are located at the binding interface with the SAM1 and SAM2 domains of Liprin-α2, respectively (Figure 6). L209 is buried inside the globular structure, which makes it plausible that the mutation affects protein folding (Figure 6). R28, D58, and G197 that did not affect CG cell survival are apart from binding interfaces (Figure 6). Consistently, cerebellar hypoplasia has not been reported in patients with these three mutations.

### 3.7. Machine Learning-Based Analysis Indicates R106P, R255C, and Y268H Mutations Disrupt Structure of Binding Interface between CASK-CaMK Domain and Liprin-α2

**Figure 7:**
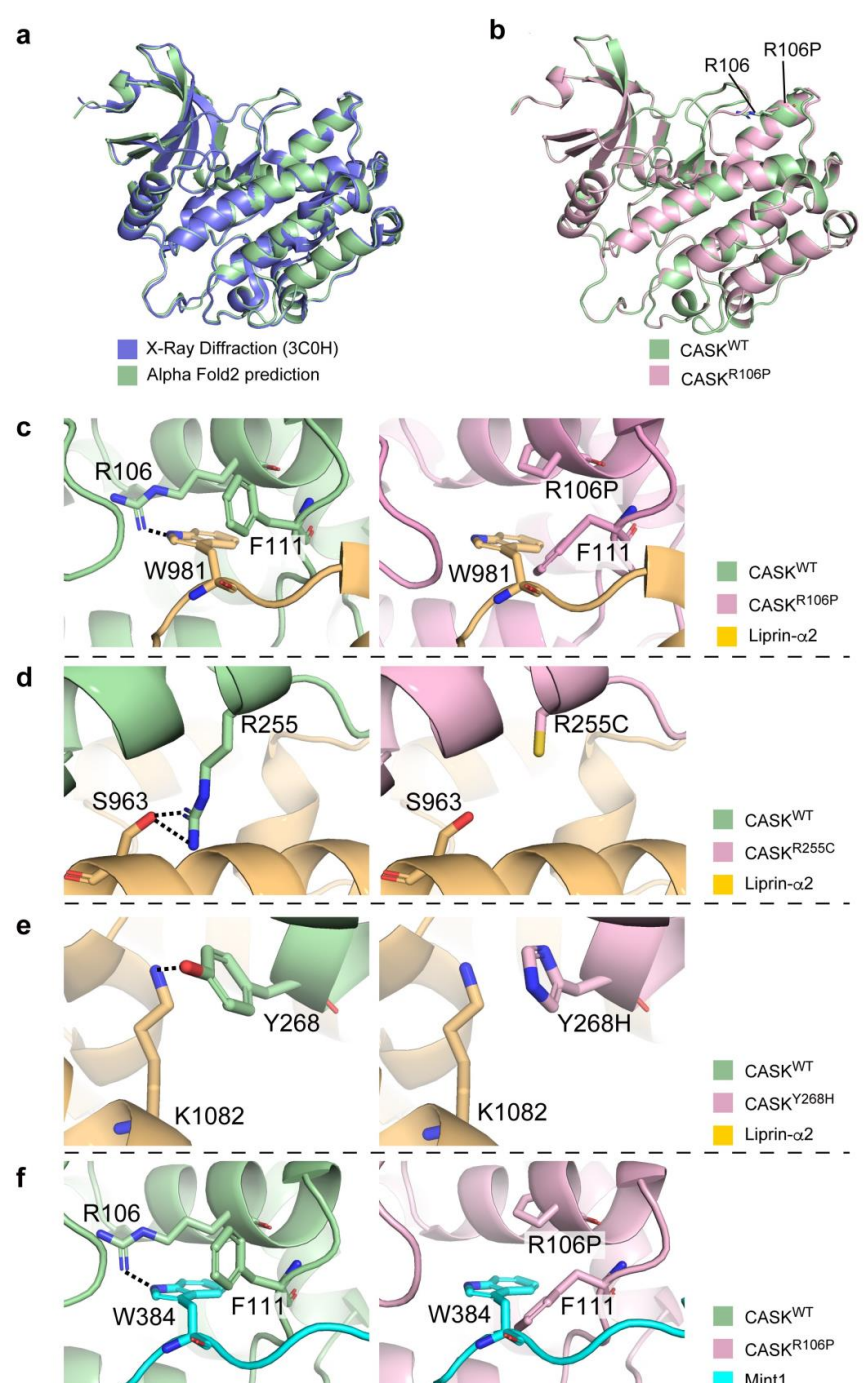
Deleterious mutations disrupt the binding interface between CASK-CaMK and Liprin-α2 predicted by AlphaFold 2.2 (DeepMind, London, UK). (**a**) Superimposed image of the 3D structure of the CASK-CaMK domain modeled by X-ray crystallography data (purple, 3C0H) and AlphaFold2.2 from the amino acid sequence (green). The AlphaFold2.2 predicted model perfectly overlaps with the X-ray crystallography. (**b**) Superimposed image of the wild-type (green) and R106P mutant (pink) CASK-CaMK domain modeled by AlphaFold2.2. The R106P mutation does not affect the overall structure of the CASK-CaMK domain. (**c**) Magnified view of the binding interface between wild-type R106 (green, left) or R106P mutant (pink, right) of CASK and W981 of Liprin-α2 (orange) modeled by AlphaFold2.2. In wild-type, W981 of Liprin-α2 is fixed between R106 and F111 of CASK where R106 and W981 form a hydrogen bond (left). The R106P mutation abolishes the hydrogen bond with W981 and alters the orientation of F111, disrupting the interaction between CASK-CaMK and Liprin-α2 (right). Liprin-α2 in the mutant panel (right) is supposed to be absent but is drawn to make the spatial orientation intelligible. (**d**) Magnified view of the binding interface between wild-type R255 (green, left) or R255C mutant (pink, right) of CASK and S968 in the SAM1 domain of Liprin-α2 (orange) modeled by AlphaFold2.2. In wild-type, R255 from CASK and S968 from Liprin-a2 form a hydrogen bond (left). The R255C mutation disrupts the hydrogen bond between these residues (right). Liprin-α2 in the mutant panel (right) is supposed to be absent but is drawn to make the spatial orientation intelligible. (**e**) Magnified view of the binding interface between wild-type Y268 (green, left) or Y268H mutant (pink, right) of CASK and K1082 in the SAM2 domain of Liprin-α2 (orange) modeled by AlphaFold2.2. In wild-type, Y268 from CASK and K1082 from Liprin-a2 form a hydrogen bond (left). The Y268H mutation disrupts the hydrogen bond between these residues (right). Liprin-α2 in the mutant panel (right) is supposed to be absent but is drawn to make the spatial orientation intelligible. (**f**) Magnified view of the binding interface between wild-type R106 (green, left) or R106P mutant (pink, right) of CASK and W384 of Mint1 (sky blue) modeled by AlphaFold2.2. In wild-type, W384 of Mint1 is fixed between R106 and F111 of CASK where R106 and W384 form the hydrogen bond (left). The R106P mutation abolishes the hydrogen bond with W384 and alters the orientation of F111, disrupting the interaction between CASK-CaMK and Mint1 (right). Mint1 in the mutant panel (right) is supposed to be absent but drawn to make the spatial orientation intelligible.

It is tempting to speculate that R106P, R255C, and Y268H mutations may disrupt binding sites to these proteins. To investigate whether these deleterious mutations affect binding properties, we utilized machine learning-based structural analysis software, AlphaFold2.2 [45,46], and built 3D structural models of the CASK-CaMK domain that contain these mutations. The sequence of CASK-CaMK domain from a CASK PDB model of 6LNM, which has the shortest additional sequence at N-terminal of CASK, was edited to introduce one of the three point mutations. We used Colab Fold v.1.4.0 [46], input a sequence of a CASK mutant as a query sequence, and obtained a PDB file of a predicted structure of the CASK-CaMK domain. To verify the accuracy of the 3D structural model created by this software, we compared the 3D structure of the wild-type CASK-CaMK domain predicted by Colab Fold v1.4.0 with another PDB model of X-ray crystallography (3C0H) on PyMOL (Schrödinger and DeLano 2020, retrieved from http://www.pymol.org/pymol (accessed on 19 December 2022)). The superimposed image of these two models perfectly overlapped (Figure 7a), suggesting that AlphaFold2.2 running on Colab Fold v1.4.0 can predict a reliable model for the CASK-CaMK domain. We compared the 3D structure of wild-type and mutant CASK-CaMK having either R106P, R255C, or Y268H mutations modeled by AlphaFold2.2. These mutations did not alter the overall structures of the CASK-CaMK domain (Figure 7b and Supplementary Figure S7).

We replaced CASK-CaMK in CASK-Liprin-α2 model (3TAC) with a predicted CASK-CaMK model on PyMOL. We investigated the effect of these mutations at the atomic level using PyMOL functions to visualize side chains and polar connections. R106 constitutes the hydrophobic pocket of the CASK-CaMK domain, where it binds to the VWV motif of Liprin-α2. R106 forms a hydrogen bond with W981 of Liprin-α2. In addition, F111 of CASK-CaMK resides appose to W981 enclosing W981 together with R106. These structures are conceivable to enhance the strength of the hydrophobic interaction with these proteins (Figure 7c left). R106P mutation withdraws the side chain of arginine and abolishes the hydrogen bond with W981. It also alters the orientation of F111, resulting in a dramatic change in the hydrophobic pocket of the CASK-CaMK domain (Figure 7c right). Similarly, W384 of Mint1 is fixed between R106 and F111 in the hydrophobic pocket of CASK-CaMK (Figure 7f left). The R106P mutation disrupts the structure of the hydrophobic pocket (Figure 7f right). These results suggest that the R106P mutation is likely to abolish the interaction with Liprin-α2 and Mint1.

R255 forms a salt bridge with S963 of Liprin-α2 (Figure 7d right). The R255C mutation abolishes this salt bridge (Figure 7d left). Similarly, Y268 forms a hydrogen bond with K1082 of Liprin-α2, and the Y268H mutation abolish it (Figure 7e). Unlike R106P mutation, R255C and Y268H solely affect the interaction with Linpin-α2, indicating that the CASK-Lipin-α2 interaction may be responsible for CG cell survival. 

**Figure 8:**
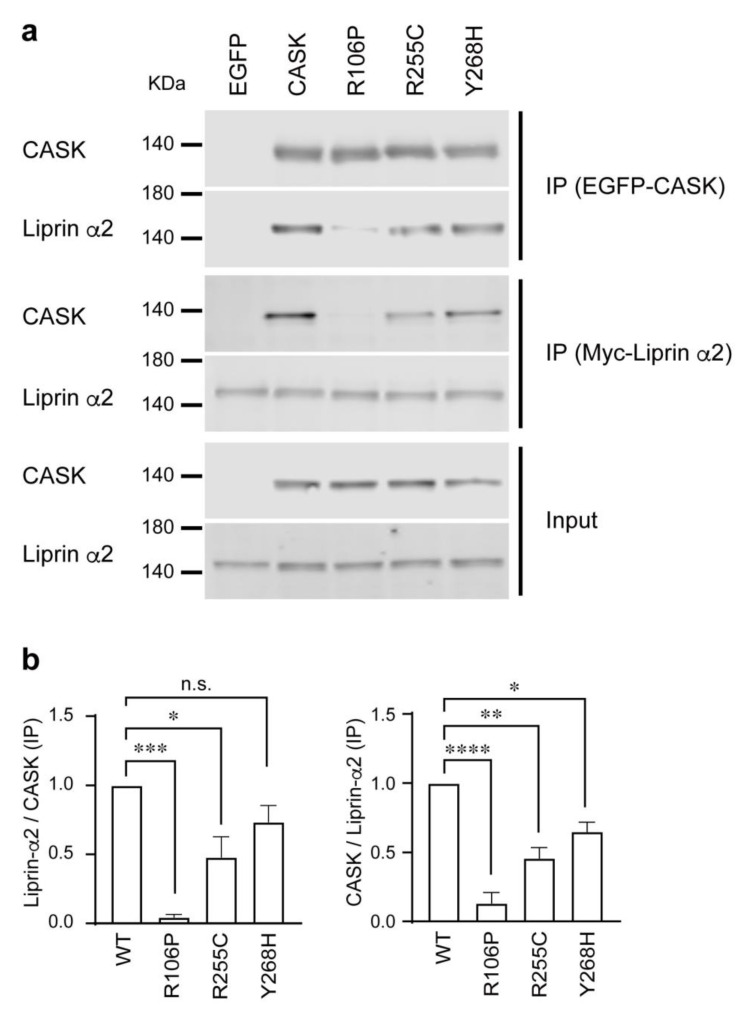
Deleterious mutations affect CASK-Liprin-α2 interaction. (**a**) Western blotting images for immunoprecipitation for analyzing binding affinity between CASK mutants and Liprin-α2. HEK293T cells were co-transfected with EGFP alone, EGFP fused wild-type or mutant CASK harboring either R106P, E255C, or Y268H mutations and Myc-tagged Liprin-α2 constructs. Cell lysates were immunoprecipitated with anti-GFP (IP EGFP-CASK) or anti-Myc antibody conjugated beads (IP Myc-Liprin). Input and immunoprecipitated proteins were subjected to Western blotting using anti-CASK or anti-Myc antibodies for measurement of binding affinity. (**b**) Binding affinity between Liprin-α2 and CASK missense mutants was measured by the co-precipitation efficiency. (**Left**) Summary graph for affinity measurement by immunoprecipitation with anti-EGFP antibody conjugated beads blotted with anti-Liprin antibody. The signal intensity was normalized by the one co-transfected with wild-type CASK. (**Right**) Summary graph for affinity measurement by immunoprecipitation with anti-Myc antibody conjugated beads blotted with anti-CASK antibody. The signal intensity was normalized by the one co-transfected with wild-type CASK. Coimmunoprecipitation experiments were repeated three times. * *p* < 0.05, ** *p* < 0.01, *** *p* < 0.001, **** *p* < 0.0001; one-way ANOVA, n.s. = not significant.

To confirm whether these mutations affect interaction with Liprin-α2, we co-transfected HEK293 cells with EGFP fused wild-type or mutant CASK harboring either R106P, R255C, or Y268H and Myc-tagged Liprin-α2 constructs and performed immunoprecipitation using anti-EGFP and anti-Myc antibodies from these lysates. R106P mutation dramatically impaired the CASK- Liprin-α2 interaction (Figure 8a,b). Not dramatically like R106P, but R255C and Y268H mutations also significantly or moderately impaired the interaction, respectively (Figure 8a,b). These results suggest that interaction with Liprin-α2 through CaMK domain is involved in the molecular mechanism by which CASK maintains CG cell survival. 

## 4. Discussion

Cerebellar hypoplasia is the most prominent phenotype in MICPCH syndrome. In this study, we investigated the molecular mechanism by which CASK deficiency induces cerebellar hypoplasia in MICPCH syndrome. For this, we used CASK KO mice as disease models. We confirmed that female CASK heterozygote KO mice replicate progressive cerebellar hypoplasia observed in MICPCH syndrome. CG cells in the CASK KO dissociated culture exhibited cell death by an apoptotic mechanism. The application of BDNF failed to rescue the CG cell death in CASK KO culture neurons, suggesting that the CG cell death is independent of Neurexin-mediated BDNF secretion. Deletion mutants lacking either the CaMK, PDZ, or SH3 domain failed to rescue the CG cell death in the CASK KO culture. Missense mutations in CaMK domain from human patients that affect CG cell survival in dissociate culture are located at the binding interface between the CASK-CaMK domain and Liprin-α2, and the mutations disrupt the structure of the binding interface predicted by the AlphaFold 2.2 program.

CASK is widely expressed in rodent brains from the embryonic stage [47], and, therefore, may also be involved in CG cell proliferation. However, studies using CASK conditional KO mice done by Srivastava et al. and Patel et al. have shown that CASK deficiency does not significantly affect the migration or proliferation of CG cells [29,30], but progressive CG cell degeneration accounts for cerebellar hypoplasia in MICPCH syndrome. They also showed that cerebellar hypoplasia in MICPCH syndrome is due to the non-cell autonomous effect of CASK deficiency [29,30]. In this study, we utilized a lentiviral gene delivery system that introduced control, Cre, or rescue constructs to more than 90% of CG cells. Thus, we cannot differentiate between the cell-autonomous and non-cell-autonomous effects of CASK due to the limitation of this approach.

It has been indicated that the transcription mechanism mediated by CASK-Tbr1 is not involved in cerebellar hypoplasia in MICPCH syndrome [25,48]. The CASK-Tbr1 interaction has been shown to be mediated by the guanylate kinase domain of CASK. The CASK construct lacking the guanylate kinase domain completely rescued CG cell death in CASK KO cultures, supporting this notion. The CASK-Neurexin interaction also requires the guanylate kinase domain of CASK. Based on this result, the CASK-Neurexin interaction may not be involved in this mechanism. Neurexin triple KO mice also show CG cell death. This is due to the non-cell autonomous effects of impaired BDNF secretion in Neurexin deficient CG cells. BDNF application failed to rescue CG cell death in CASK KO cultured neurons. Missense mutations affecting the CASK-Neurexin interaction have been identified in patients with cerebellar hypoplasia [42]. In this study, we focus on CG cell survival in the dissociated culture system and simply monitor CG cell death. We could not completely exclude the contribution of Neurexins in this disorder. 

We identified mutations that failed to rescue CG cell death in CaMK, PDZ, or SH3 domains. Together with the deletion mutant rescue experiments, all these domains may be independently or cooperatively involved in the mechanism of CG cell survival. In this study, we focused on mutations in CaMK domain for the structural analysis. The L209P mutation affects protein expression of CASK, and this can explain the CG cell death phenotype due to the loss-of-function effect of CASK. The R28L, D58E, and G197R mutations are also in the CaMK domain but completely rescued CG cell death. These sites are isolated from the binding interface with other known proteins. R106P is located in the hydrophobic pocket that constitutes a common binding site with hydrophobic motifs of Liprin-α2 (V980, W981, and V982) and Mint1 (I383, W384, and V385). The R106P mutation abolishes hydrogen bonds with W981 of Liprin-α2 or W384 of Mint1. This affects the strength of hydrophobic interaction between these proteins, as well as the shape of the hydrophobic pocket. R255 and Y268 form hydrogen bonds between S963 of SAM1 and K1082 of SAM2 domain of Liprin-α2, respectively [10]. The R255C and Y268H mutations eliminate these hydrogen bonds, likely disrupting the interaction with Liprin-α2. These sites are solely involved in the binding with Liprin-α2. These results indicate that the disruption of CASK-Liprin-α2 interaction may be involved in the mechanism underlying CG cell death we observed in this study.

Although the in silico analysis provides valuable information, we must acknowledge the limitations of the protein structure prediction using AlphaFold 2.2. It utilizes pre-existing PDB models as a template for machine learning, which may have influenced the overall structure of predicted models. Some mutations may affect the folding of the domain, but this possibility may have been ignored in our analysis. Furthermore, these mutations may impact the conformation of the entire CASK protein. Since the PDB model that includes all CASK domains that can be used as a template is unavailable at present, we omitted exploring these effects in this study. 

Although both CASK and Liprin-α2 are widely expressed across brain areas, cell death is observed primarily in CG cells. The cell-type-specific mechanism underlying this phenomenon is currently unknown. Further studies are required to underscore how CASK-Liprin-α2 interaction contributes to CG cell survival in a cell-type-specific manner.

## 5. Conclusions

We investigated the molecular mechanism that causes cerebellar hypoplasia in MICPCH syndrome using CASK KO mice and found that missense mutations in the CaMK domains identified from human patients affect CG cell survival in the dissociated culture system. These mutations are located at the binding interface with Liprin-α2, suggesting that the CASK-Liprin-α2 interaction may be involved in the pathophysiology of cerebellar hypoplasia in MICPCH syndrome.

## Data Availability

All datasets used and/or analyzed during the current study are available from the corresponding authors on reasonable request.

## Supplementary Materials

The following supporting information can be downloaded at: https://www.mdpi.com/article/10.3390/cells12081177/s1.
