# Supplementary material for: Structural Analysis Implicates CASK-Liprin-α2 Interaction in Cerebellar Granular Cell Death in MICPCH Syndrome
Source: Cells. 2023 Apr 18;12(8):1177. doi: 10.3390/cells12081177 (PMC10136522; doi:10.3390/cells12081177)
Supplement: Supplementary file 1 [file cells-12-01177-s001.zip › cells-2058969-supplementary/Supplementary Figures.pdf]

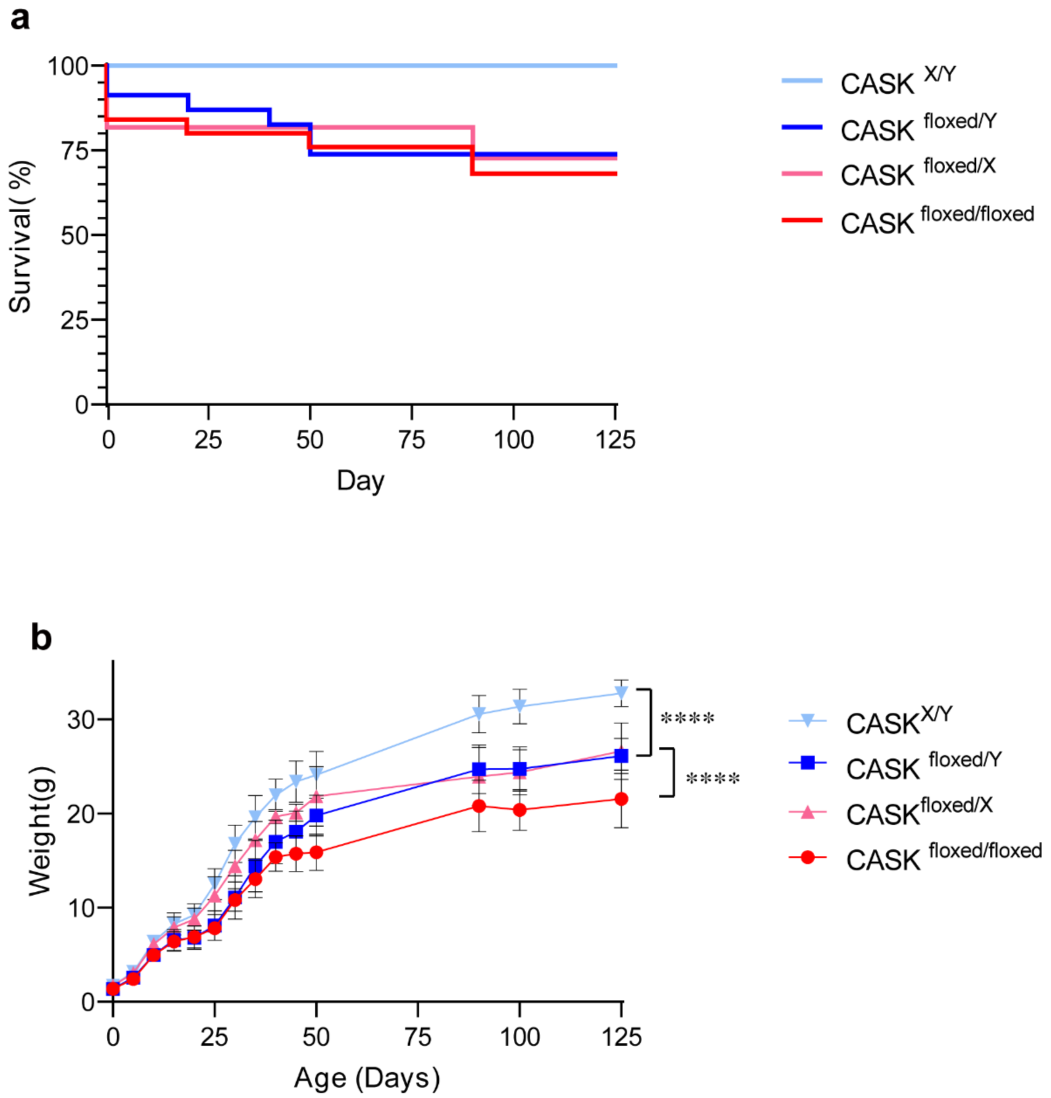

Figure S1. Survival, the body weight of CASK*floxed/floxed* mice. a, Survival of CASK floxed mice obtained from interbreeding with heterozygote CASK floxed mice (CASK floxed/Y and CASK floxed/X). **b**, Plot of the body weight (means ± SEMs) of littermate male WT (X/Y), heterozygous female (floxed/X), and fCASK (floxed/Y, floxed/floxed).

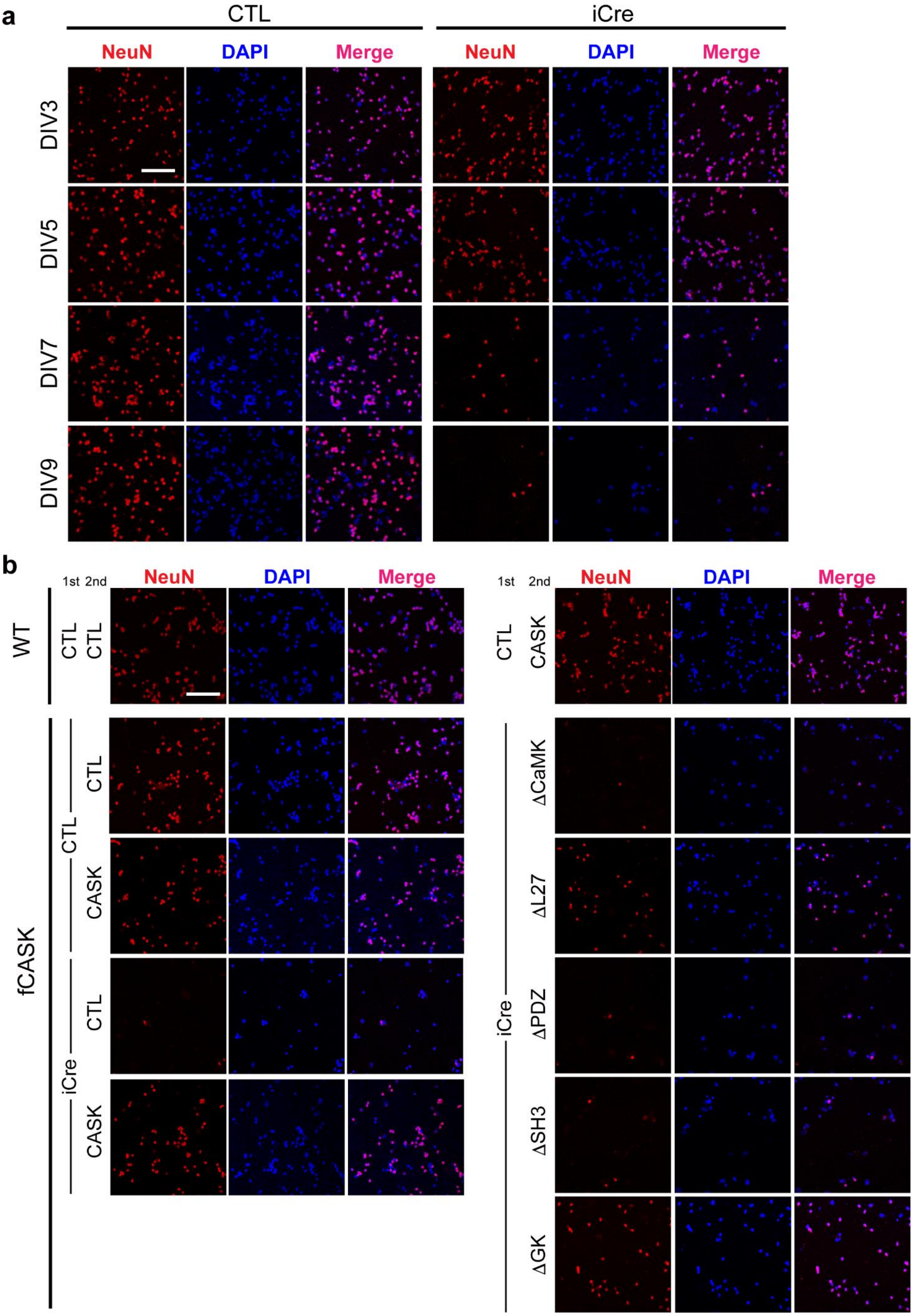
a
CTL
iCre
NeuN
DAPI
Merge
NeuN
DAPI
Merge
DIV3
DIV5
DIV7
DIV9
b
1st
2nd
NeuN
DAPI
Merge
WT
CTL
CTL
fCASK
CTL
CTL
CASK
iCre
CTL
CASK
1st
2nd
NeuN
DAPI
Merge
CTL
CASK
iCre
ΔCaMK
ΔL27
ΔPDZ
ΔSH3
ΔGK

Figure S2. NeuN staining of cultured CGCs from wild-type and CASK floxed mice infected with lentivirus-iCre and/or CASK rescue constructs. a, Cultured fCASK CGCs are infected with lentivirus-control (CTL) or lentivirus-iCre (iCre) fixed and stained at DIV3, 5, 7, and 9. b, Cultured CGCs from WT and fCASK mice infected with lentivirus-control (CTL) or lentivirus-iCre (iCre) together with lentivirus-CASK (full-length or deletion mutants) stained with an antibody against NeuN (red) and DAPI (blue) at DIV9. Scale bar = 100 μm.

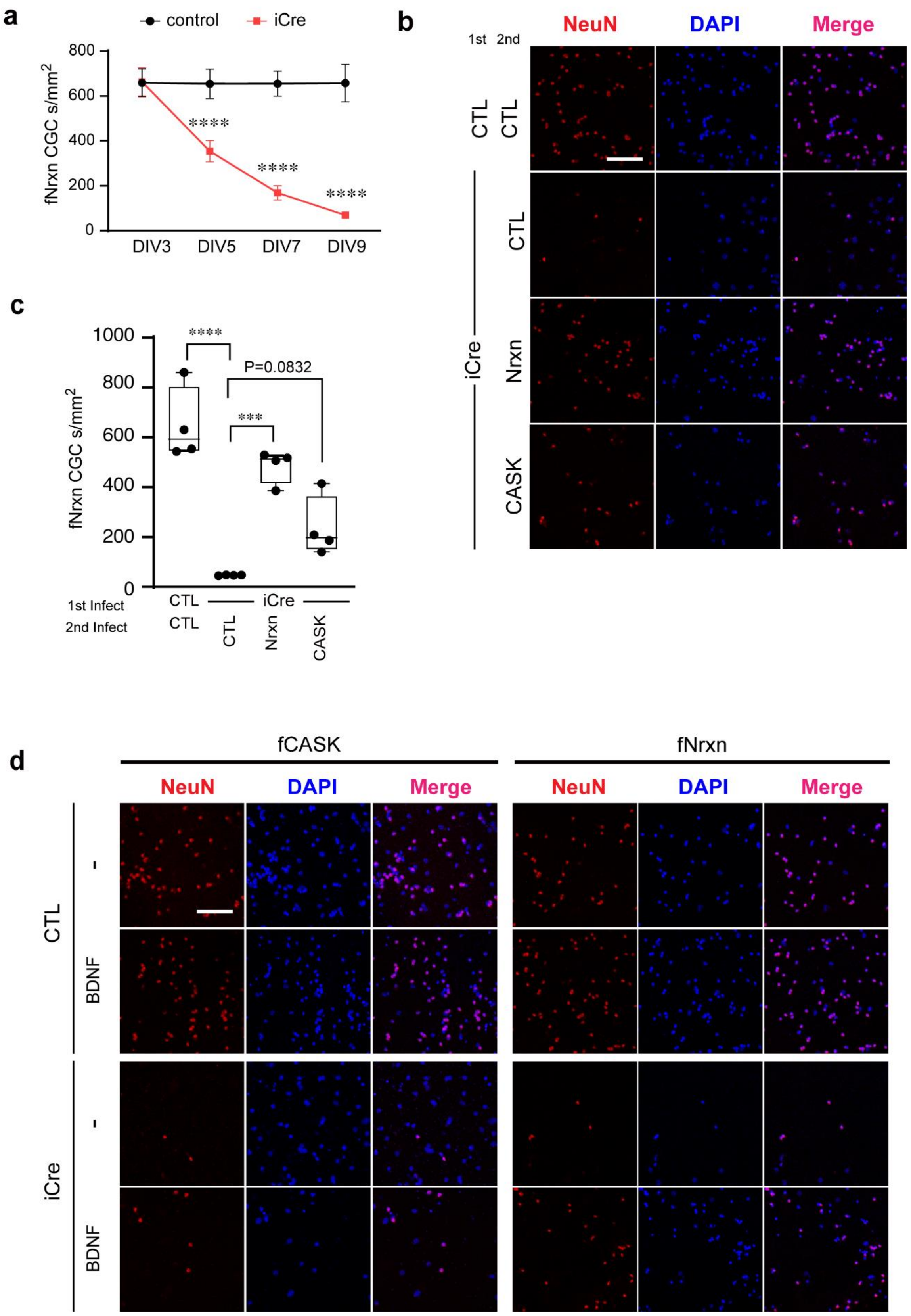

Figure S3. BDNF does not rescue the cell survival defects in cultured CASK KO CGCs. **a**, Cultured fNrxn CGCs are infected with lentivirus-iCre. The cells are stained with an antibody against NeuN and DAPI. The number of NeuN-positive CGCs is quantified. The values are averages of 3 points for each well and 6 wells for each sample. ****P<0.0001; two-way ANOVA. **b**, Cultured fNrxn CGCs are infected with lentivirus-iCre together with lentivirus-Nrxn or lentivirus-CASK. The cells are stained with an antibody against NeuN (red) and DAPI (blue). Scale bar = 100 μm. **c**, Quantification of the number of NeuN-positive CGCs in **b**. The values are averages of 3 points for each well and 4 wells for each sample. Co-infection with lentivirus-CASK fails to rescue the CGC cell-death. **d**, BDNF is added to cultured CGCs prepared from fCASK or fNrxn mice infected with lentivirus-control (CTL) or lentivirus-iCre (iCre). The cells are stained with the antibody against NeuN (red) and DAP (blue). Scale bar = 100 μm.

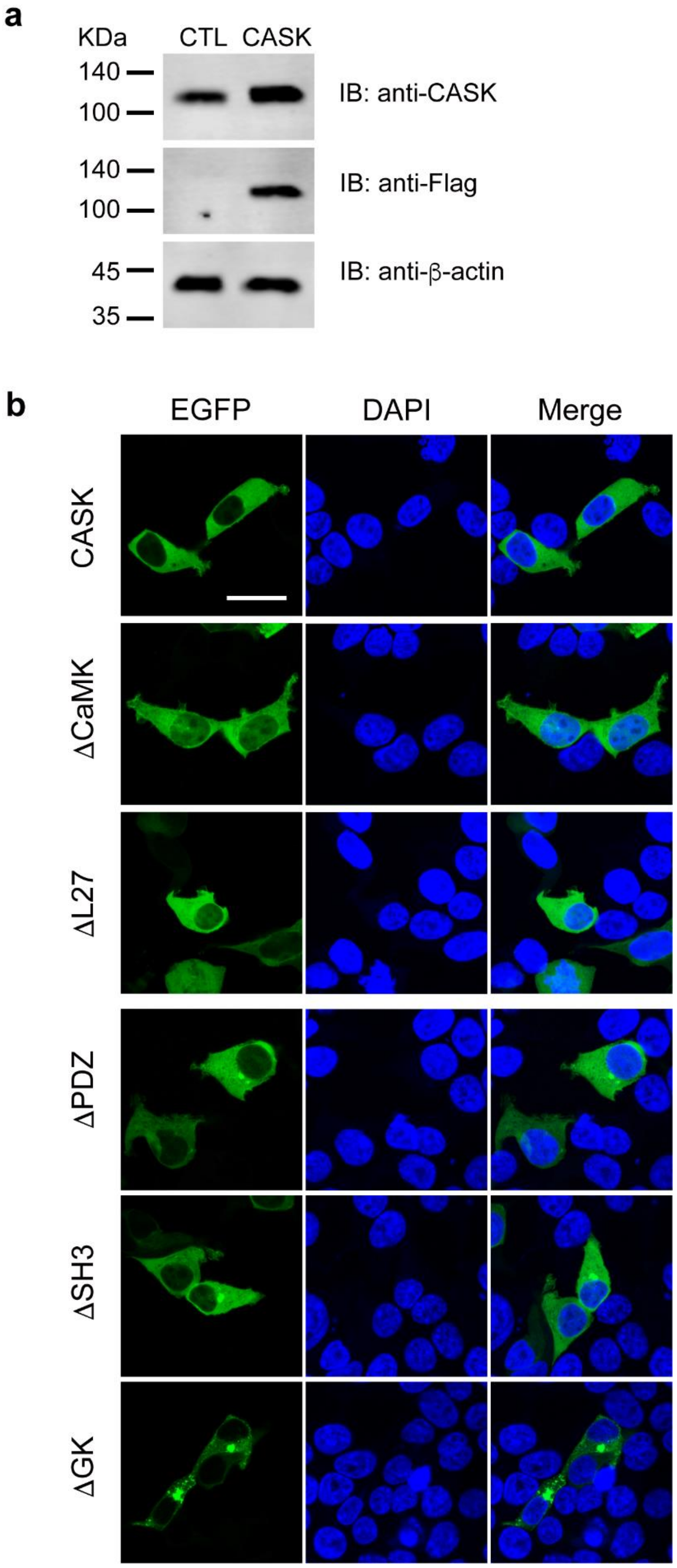

Figure S4. Expression of the deletion mutant of CASK. a, Western blot images for examining the expression of Flag-tagged full-length CASK protein in cultured CGCs introduced with lentiviral infection. Cell lysates from lentivirus-control (CTL) or lentivirus-Flag-CASK (CASK) infected CGCs are blotted by antibodies against CASK, Flag, and β-actin. **b**, Expression of full-length and deletion mutants of CASK in HEK293T. HEK293T cells transfected with EGFP-fused full-length or deletion mutants of CASK expression vectors are fixed 1 day after transfection and stained with DAPI. Scale bar = 20 μm.

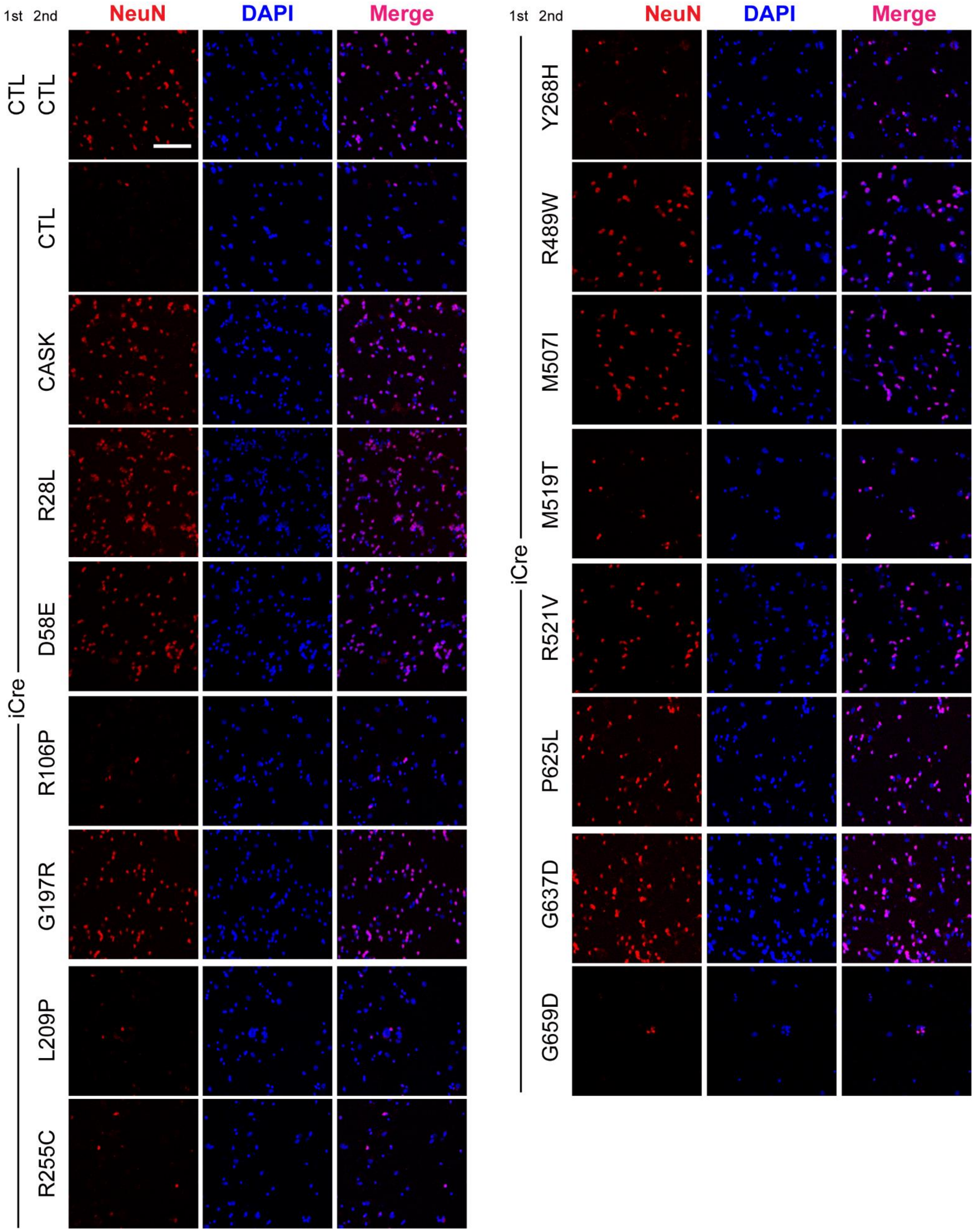

Figure S5. NeuN staining of CGCs infected with lentivirus-iCre and/or CASK missense mutations. Cultured CGCs are infected with lentivirus-iCre together with lentivirus-CASK (WT or missense mutants). The cells are stained with the antibody against NeuN (red) and DAPI (blue). Scale bar = 100 μm.

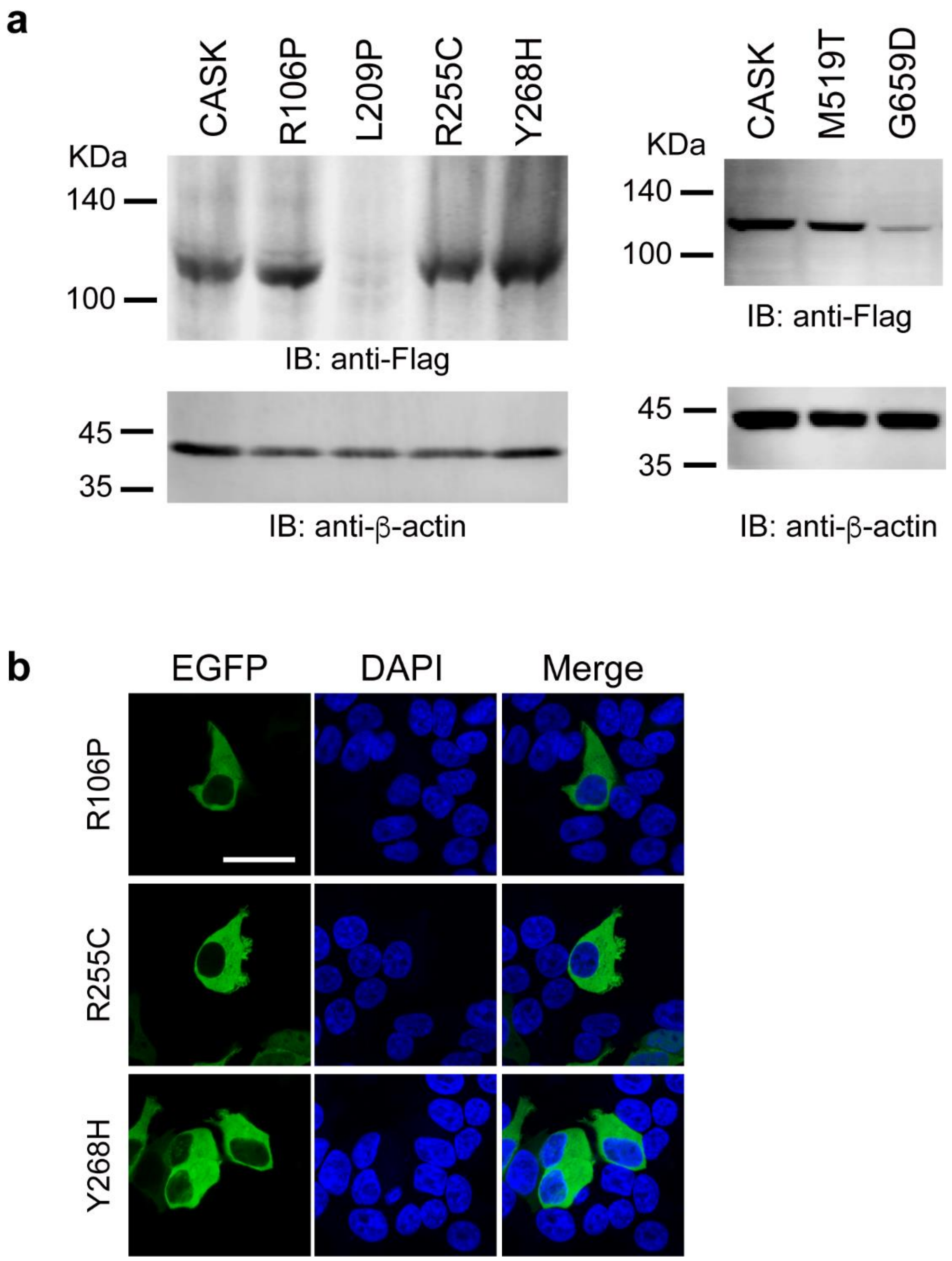

Figure S6. Expression of missense mutant of CASK. a, Western blot analysis of the expression of missense mutants of CASK. Cell lysates from WT CGCs infected with lentivirus expressing Flag-tagged CASK variants are blotted with anti-Flag and β-actin antibodies. **b**, Expression of missense mutants of CASK in HEK293T. HEK293T cells expressing EGFP fused CASK having R106P, R255C, or Y268H mutations are fixed 1day after transfection and stained with DAPI. Scale bar = 20 μm.

a

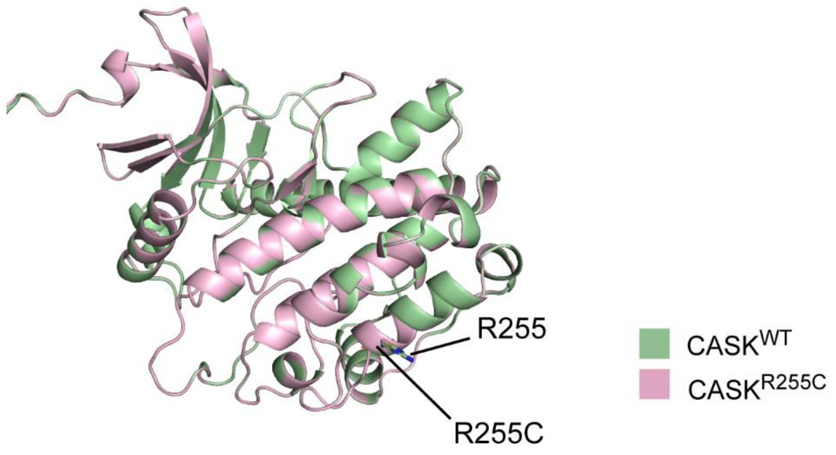

b

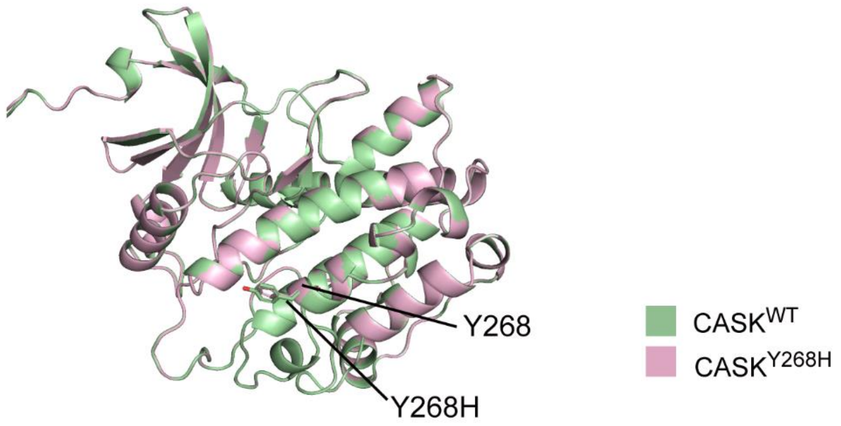

Figure S7. **a**, Superimposed image of wild-type (Green) and R255C mutant (Pink) CASK CaMK domain modeled by AlphaFold2.2. **b**, Superimposed image of wild-type (Green) and Y268H mutant (Pink) CASK CaMK domain modeled by AlphaFold2.2.
